# Mapping of Schistosomiasis and Soil-Transmitted Helminthiasis in the Regions of Centre, East and West Cameroon

**DOI:** 10.1371/journal.pntd.0001553

**Published:** 2012-03-06

**Authors:** Louis-Albert Tchuem Tchuenté, Romuald Isaka Kamwa Ngassam, Laurentine Sumo, Pierre Ngassam, Calvine Dongmo Noumedem, Deguy D'or Luogbou Nzu, Esther Dankoni, Christian Mérimé Kenfack, Nestor Feussom Gipwe, Julie Akame, Ann Tarini, Yaobi Zhang, Fru Fobuzski Angwafo

**Affiliations:** 1 National Programme for the Control of Schistosomiasis and Intestinal Helminthiasis, Ministry of Public Health, Yaoundé, Cameroon; 2 Laboratory of Parasitology and Ecology, University of Yaoundé I, Yaoundé, Cameroon; 3 Centre for Schistosomiasis and Parasitology, Yaoundé, Cameroon; 4 Helen Keller International, Yaoundé, Cameroon; 5 Helen Keller International, Regional Office for Africa, Dakar, Senegal; 6 Ministry of Public Health, Yaoundé, Cameroon; Centre Suisse de Recherches Scientifiques, United States of America

## Abstract

**Background:**

Schistosomiasis and soil-transmitted helminthiasis (STH) are widely distributed in Cameroon. Although mass drug administration (MDA) of mebendazole is implemented nationwide, treatment with praziquantel was so far limited to the three northern regions and few health districts in the southern part of Cameroon, based on previous mapping conducted 25 years ago. To update the disease distribution map and determine where treatment with praziquantel should be extended, mapping surveys were conducted in three of the seven southern regions of Cameroon, i.e. Centre, East and West.

**Methodology:**

Parasitological surveys were conducted in April–May 2010 in selected schools in all 63 health districts of the three targeted regions, using appropriate research methodologies, i.e. Kato-Katz and urine filtration.

**Principal Findings:**

The results showed significant variation of schistosomiasis and STH prevalence between schools, villages, districts and regions. *Schistosoma mansoni* was the most prevalent schistosome species, with an overall prevalence of 5.53%, followed by *S. haematobium* (1.72%) and *S. guineensis* (0.14%). The overall prevalence of schistosomiasis across the three regions was 7.31% (95% CI: 6.86–7.77%). The prevalence for *Ascaris lumbricoides* was 11.48 (95% CI: 10.93–12.04%), *Trichuris trichiura* 18.22% (95% CI: 17.56–18.90%) and hookworms 1.55% (95% CI: 1.35–1.78%), with an overall STH prevalence of 24.10% (95% CI: 23.36–24.85%) across the three regions. STH was more prevalent in the East region (46.57%; 95% CI: 44.41–48.75%) in comparison to the Centre (25.12; 95% CI: 24.10–26.17%) and West (10.49%; 95% CI: 9.57–11.51%) regions.

**Conclusions/Significance:**

In comparison to previous data, the results showed an increase of schistosomiasis transmission in several health districts, whereas there was a significant decline of STH infections. Based on the prevalence data, the continuation of annual or bi-annual MDA for STH is recommended, as well as an extension of praziquantel in identified moderate and high risk communities for schistosomiasis.

## Author Summary

Schistosomiasis and soil-transmitted helminthiasis (STH) are a major public health problem in Cameroon. The national control strategy of these diseases was based on historical data collected 25 years ago, which might be outdated in some situations due to several factors including control activities, improved or degraded sanitation and hygiene, socio-economic improvement and disease transmission dynamics. To help planning, improving control strategies and evaluation of control activities, there was a need to update the distribution of schistosomiasis and STH. We conducted parasitological surveys in three regions of Cameroon, i.e. Centre, East and West. Our results showed a significant decrease of STH infection prevalence and intensities in all these three regions, in comparison to previous mapping data, with an overall decline of prevalence from 81.1–93% to 10.5–46.6%. These results show the positive impact of annual deworming campaigns, and illustrate the progressive success of the national programme for the control of schistosomiasis and STH in Cameroon. Furthermore, our results showed an increase of the number of high transmission foci of schistosomiasis, and allowed identifying new health districts requiring mass treatment with praziquantel, and those where deworming should be reinforced.

## Introduction

Recent years have witnessed an increased interest in the control of neglected tropical diseases (NTDs), and today there exists a global momentum for the control of these diseases, as well as an unprecedented opportunity for cost-effective action, through an integrated control [1]–[5]. Interest in the integrated control of NTDs is currently at an all-time high, due in part to new funding committed by a number of governmental and non-governmental donors, high-level political commitment in the endemic countries, and the existence of donated anthelminthic drugs which can be safely co-administrated and used in a coordinated way to address these scourges [6]–[8]. Four of these diseases are mainly controlled through the ‘preventive chemotherapy’ intervention, i.e. schistosomiasis, soil-transmitted helminthiasis (STH), onchocerciasis and lymphatic filariasis, according to the World Health Organization (WHO) recommendations [4]. Schistosomiasis and STH occur throughout the developing world and remain a major public health problem in the poorest communities with enormous consequences for development. Praziquantel is the sole drug for treatment and morbidity control of schistosomiasis in sub-Saharan Africa. Control of STH uses two main drugs, i.e. albendazole or mebendazole. Based on infection prevalence, communities can be classified into low-risk (<10% for schistosomiasis and <20% for STH), moderate-risk (≥10% but <50% for schistosomiasis and ≥20% but <50% for STH) and high-risk (≥50% for both) categories according to the WHO disease specific thresholds, and this classification is used to determine the appropriate treatment regimen as specified in the WHO guidelines [4].

In Cameroon, it is estimated that more than 5 million people are at risk of infection with schistosomiasis, and 2 million persons are currently infected [9]. STHs are widely distributed all over the country, and it is estimated that more than 10 million people are infected with intestinal worms [9]. The national epidemiological survey conducted in 1985–1987 showed the occurrence of three species of schistosomes: *Schistosoma haematobium*, *S. mansoni* and *S. guineensis* (formerly *S. intercalatum* Lower Guinea strain [10], [11]); and three major species of STH: *Ascaris lumbricoides*, *Trichuris trichiura* and *Necator americanus*. The highest transmission levels of schistosomiasis occurred in the savannah areas of the northern Cameroon, whereas STHs were more prevalent in the southern forest part of the country [12]–[14]. School-aged children are the most infected, and polyparasitism is very frequent; with a high proportion of children carrying at least 2 species of parasites [15].

Cameroon adopted a strategic plan for the control of schistosomiasis and STH in 2004. Starting with very limited budget, the control programme gradually mobilized national and international partners to enable a rapid scaling-up of activities to encompass all ten regions in 2007. Since then, national deworming campaigns were implemented annually. School-aged children were treated with mebendazole nationwide, whereas praziquantel was distributed only in high endemic areas for schistosomiasis [16]. Interestingly, the Government of Cameroon recently moved into an integrated approach for the control of NTDs, including co-implementation of different control interventions and co-administration of several drugs, i.e. praziquantel, ivermectin, mebendazole and albendazole. This integrated approach is the basis for cost-effectiveness and streamlined efficiency. Since 2009, Cameroon receives assistance from the United States Agency for International Development (USAID) through its NTD Control Program to facilitate integration of national programs and support mass drug administration (MDA) [17].

Because knowing the distribution of the targeted NTDs is essential for developing an adequate implementation strategy and types of drug co-administrations, one of the efforts of the USAID's NTD control program in Cameroon was focused on updating the disease-distribution information. Hence, efforts were made to support on-the-ground activities to map the disease distribution where sufficient information was not available. Indeed, the baseline data for schistosomiasis and STH in Cameroon were collected 25 years ago [12], [13]. It is well known that the transmission of these diseases is dynamic over time, particularly after years of treatment and other health interventions [18]. Therefore, epidemiological surveys were scheduled in the different regions of Cameroon in order to update the distribution and the level of endemicity of schistosomiasis and STH to facilitate the planning of implementation strategies in these regions. The first study phase targeted three of the ten regions of Cameroon, i.e. Centre, East and West. The present paper reports the outcome of the mapping exercises, compares the current situation with the baseline data from 1980s, and provides recommendations for the control of schistosomiasis and STH in these regions.

## Methods

### Ethical statement

The study was approved by the National Ethics Committee of Cameroon (Nr 082/CNE/DNM/09), and was a public health exercise through the Ministry of Public Health and the Ministry of Basic Education. Parasitological surveys were conducted in schools with the approval of the administrative authorities, school inspectors, directors and teachers. Information about the national programme for the control of schistosomiasis and STH, and the objectives of the study were explained to the schoolchildren and to their parents or guardians from whom written informed consent was obtained. Children willing to participate were registered. Each child was assigned an identification number and data collected were entered in a database. No identification of any children can be revealed upon publication. Children were treated during the MDA campaign implemented by the national control programme.

### Study area

Cameroon is divided up into a three-tiered system including 10 regions at the first level, 58 divisions (*departments*) at the second level, and 360 sub-districts (*arrondissements*) at the third level. The population of Cameroon is estimated to be 19,406,100 inhabitants in 2010. Population density shows marked variation across the country, ranging from a mean of 7.4 inhabitants/km^2^ in the East region to 141.5 inhabitants/km^2^ in the Littoral region. School-aged children account for 28% of the country population and are estimated at 5,433,708 [19]. The health system in Cameroon is decentralized and organized into central, regional and district levels. There are 179 health districts. The three regions targeted for mapping, i.e. Centre, West and East are located in the southern forest area of the country. These regions are subdivided in 29, 14 and 20 health districts, respectively.

### Sampling and data collection

A stratified random-cluster sampling procedure, with the 5^th^ grade as the basic sampling unit, was used in the previous mapping of schistosomiasis and STH in Cameroon, conducted in 1985–1987 [12], [20]. In order to assess the current levels of infections and to compare the data with previous ones, the schools were selected using the list of villages and schools previously investigated, the ecological zones and the risk factors for schistosomiasis transmission [21], [22]. Selection was made so that all health districts in the three targeted regions of Cameroon were covered spatially. Due to financial limitations, an average of four primary schools (proportional to the district's size and population density) was selected per health district. The geographical co-ordinates of each of the sampled schools were recorded with global positioning system (GPS) devices. The study was conducted in April–May 2010.

In the 1985–1987 study, a 10 ml urine sample and a single Kato-Katz slide were examined for schistosome and STH infections [12], [20]. In the current study, in each school, urine and stool samples were collected from 50 children selected randomly in the upper classes, approximately half boys and half girls. Children were preferentially selected from the 5^th^ grade, and then in other grades where the number of children in the 5^th^ grade was fewer than 50. The samples were collected in 60 mL plastic screw-cap vials, between 10.00 and 14.00 hours. The samples were preserved with sodium azide [12], [20] and transported to the *Centre for Schistosomiasis & Parasitology* in Yaoundé for examination. In the laboratory, each urine sample was agitated to ensure adequate dispersal of eggs, 10 mL of urine were filtered through a Nucleopore® filter, and the filters were examined by microscopy for the presence of schistosome eggs. Stool samples were examined by a single thick smear technique using a 41.7 mg Kato-Katz template. Each Kato slide was read twice; immediately after slide preparation for hookworm eggs, and the following day for schistosome and other STH eggs. Parasitic infections were recorded; number of eggs for each parasite was counted; and intensity of infection was calculated and expressed as eggs per gram of feces (epg) or eggs per 10 ml of urine (egg/10 ml).

### Data analysis

The different parasitological data were analyzed by the epidemiological unit of the *Centre for Schistosomiasis & Parasitology* using appropriate statistical tests and methods. The data were subsequently exported into SPSS (IBM, Version 19) for statistical analysis. The Complex Samples Crosstabs procedure was used for calculating the prevalence and the Descriptives procedure was used for calculating the intensity of infections, taking into account the cluster nature of schools with districts as strata and schools as clusters and including the finite population correction assuming equal probability sampling without replacement. Sample weighting was applied for each district according to the ratio of the proportionally expected number of schools to be surveyed and the number of actually surveyed schools in each district assuming similar number of children in each school [23]. The 95% confidence intervals (CIs) for prevalence were calculated using the Wilson score method without the continuity correction after adjusting for sample weighting [24]. Arithmetic mean intensities of infection with 95% CIs for different parasite species were calculated including all children examined [25]–[27]. The Chi-square test using the Complex Samples Crosstabs procedure was used to investigate the relationship between prevalence of infections and sex, age groups, districts and regions, and the Complex Samples Logistic regression procedure was used to compare the differences in prevalence between 1985–1987 and 2010. The Kruskal-Wallis test was used to compare the differences in intensities of infections. The levels of endemicity of schistosomiasis and STH and the degrees of intensity of individual infections were categorized according to the WHO recommendations [4], [28]. A geographical information system (GIS) software ArcGIS (ESRI Inc., Version 9.2) was used to plot the point prevalence of the infections for each surveyed school on a map.

## Results

A total of 244 schools were surveyed: 118 in the Centre region, 67 in the East region and 59 in the West region. A total of 12 594 pupils aged 2–23 years old (6251 males and 6343 females) from these 244 schools were registered and included in the study. Of these children registered, 12 486 (99.14%) provided urine samples and 12 243 (97.21%) provided stool samples. The mean age (± standard deviation) of children examined was 11.30±1.98 years (male: 11.45±2.0 and female: 11.15±1.94).

### Schistosomiasis

#### Prevalence

Table 1 summarizes the survey results for the different parasite species in each region. The results are shown as prevalence and intensity of infections together with 95% CI. Detailed analysis of the results showed significant variation of infection prevalence between schools, villages, districts and regions (*P<0*.001).

**Table 1:** Prevalence and intensity of infections in school children in the three surveyed regions in Cameroon.

	*S. haematobium*	*S. guineensis*	*S. mansoni*	*A. lumbricoides*	Hookworm	*T. trichiura*
**Prevalence (%)**						
Overall	1.72 (1.52–1.97)*	0.14 (0.08–0.21)	5.53 (5.14–5.94)	11.48 (10.93–12.04)	1.55 (1.35–1.78)	18.22 (17.56–18.90)
By region						
Centre	1.11 (0.89–1.38)	0.21 (0.12–0.34)	9.49 (8.82–10.21)	10.48 (9.78–11.24)	2.67 (2.31–3.08)	18.60 (17.69–19.54)
East	2.01 (1.48–2.70)	-	0.08 (0.03–0.36)	28.81 (26.90–30.84)	0.52 (0.30–0.97)	37.52 (35.43–39.63)
West	2.69 (2.22–3.24)	0.08 (0.03–0.23)	1.36 (1.03–1.76)	4.13 (3.54–4.80)	0.12 (0.06–0.30)	7.41 (6.63–8.29)
By sex						
Male	2.14 (1.81–2.52)	0.09 (0.04–0.21)	6.92 (6.32–7.57)	12.43 (11.63–13.26)	1.62 (1.34–1.97)	19.40 (18.44–20.39)
Female	1.31 (1.06–1.61)	0.18 (0.11–0.33)	4.14 (3.67–4.65)	10.54 (9.8–11.31)	1.48 (1.21–1.81)	17.05 (16.15–18.00)
**Intensity of infection (epg)** **						
Overall	2.46 (0.06–4.86)	0.23 (0.03–0.44)	33.24 (21.11–45.36)	1418.23 (1103.09–1733.37)	4.78 (2.85–6.72)	199.15 (129.93–268.37)
0 epg (%)	98.06 (97.80–98.29)	99.88 (99.80–99.93)	95.23 (94.83–95.59)	85.34 (84.70–85.96)	98.50 (98.26–98.70)	78.08 (77.33–78.81)
Light (%)	1.22 (1.04–1.43)	0.06 (0.03–0.12)	2.25 (2.00–2.52)	7.71 (7.25–8.21)	1.47 (1.27–1.70)	17.40 (16.73–18.09)
Moderate (%)	-	0.05 (0.02–0.11)	1.31 (1.12–1.52)	6.08 (5.66–6.52)	0.02 (0.00–0.06)	4.04 (3.70–4.41)
Heavy (%)	0.72 (0.59–0.89)	0.02 (0.00–0.06)	1.22 (1.04–1.43)	0.86 (0.71–1.05)	0.02 (0.00–0.06)	0.48 (0.37–0.62)
By region						
Centre	0.17 (0.00–0.38)	0.40 (0.02–0.77)	61.04 (39.36–82.73)	1086.02 (746.77–1425.27)	8.55 (5.02–12.08)	113.04 (71.90–154.18)
East	2.03 (0.10–3.96)	-	0.12 (0.00–0.27)	4810.82 (3223.13–6398.51)	1.16 (0.00–2.81)	843.21 (434.86–1251.56)
West	6.86 (0.00–14.82)	0.06 (0.00–0.16)	1.22 (0.26–2.18)	218.26 (105.41–331.12)	0.06 (0.00–0.14)	11.92 (7.49–16.35)
By sex						
Male	4.05 (0.00–8.44)	0.21 (0.00–0.56)	41.69 (24.74–58.64)	1378.60 (1025.70–1731.50)	4.11 (2.90–5.32)	203.60 (134.52–272.69)
Female	0.87 (0.13–1.62)	0.25 (0.05–0.46)	24.82 (12.55–37.09)	1457.71 (1049.47–1865.94)	5.46 (1.89–9.02)	194.72 (121.41–268.02)

Note:

*Figures in brackets represent the 95% confidence intervals.

**eggs/10 ml urine for *S. haematobium* intensity of infection; light, moderate and heavy infections was categorized according to the WHO recommendations (Ref #26); *S. guineensis* infection was categorized using the recommendations for *S. mansoni*.

**Figure 1:**
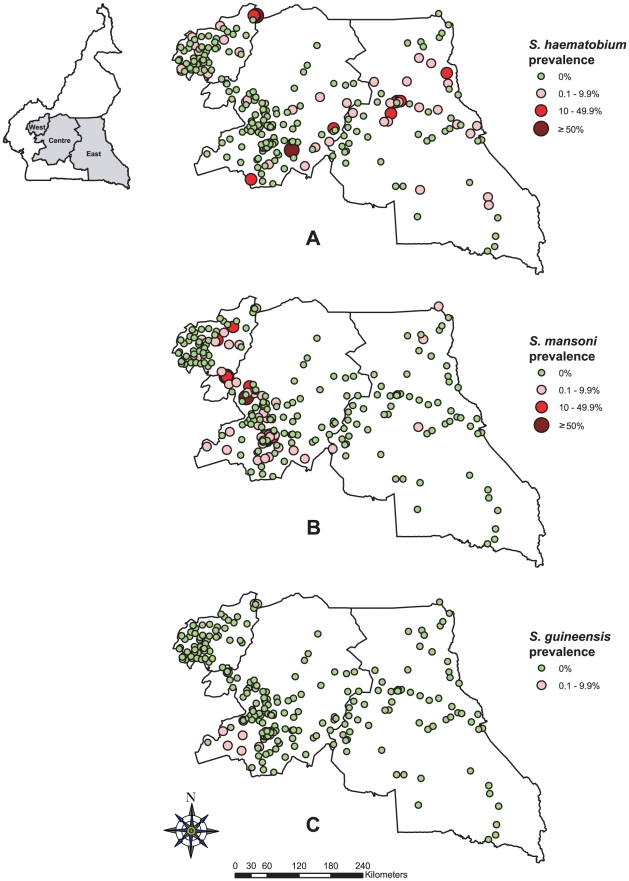
Prevalence of schistosomiasis by school in the three surveyed regions of Cameroon. (A) *S. haematobium*, (B) *S. mansoni*, and (C) *S. guineensis*.

*S. mansoni* was the most prevalent schistosome species. Infected children were found in 60 of the 244 schools investigated, with an average prevalence of 5.53% ranging from 0% to 66.36% across the three regions. *S. haematobium* was found in 50 schools with an average prevalence of 1.72% ranging from 0% to 95.92% across the three regions. The highest prevalence of schistosomiasis, 95.92% for *S. haematobium*, was found in the Malantouen health district, West region. *S. guineensis* was found in 11 schools, with relatively low prevalence of 0.14% varying from 0% to 6%. The point prevalence distribution of schistosomiasis in all surveyed schools is shown in Figure 1. There was a significant difference of schistosome infections between schools and villages. The majority of schools were negative for schistosomiasis.

Among the three regions surveyed, *S*. *mansoni* infection was highest in the Centre region with an average prevalence of 9.49%, followed by the West region (1.36%) and the East region (0.08%) (Table 1). *S. haematobium* infection prevalence was 2.69% in the East region, 2.01% in the West region and 1.11% in the Centre region. Low level of *S. guineensis* infection was also found in the Centre and West regions. The overall prevalence of schistosomiasis (including all three species) across the three regions was 7.31%: 10.71% in the Centre region, 3.98% in the West region and 2.05% in the East region.

**Figure 2:**
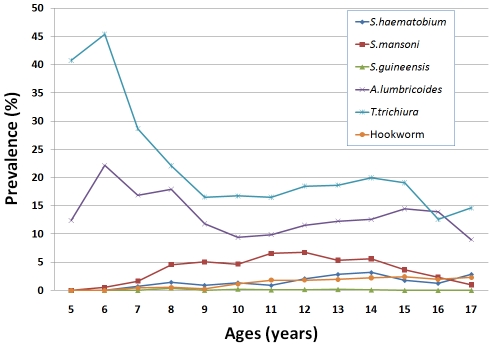
Prevalence of schistosomiasis and STH by age in the three surveyed regions in Cameroon. A few outliers below 5 or above 17 years old are not plotted.

More boys were infected with *S. mansoni* and *S. haematobium*, as well as the overall schistosome infections, than girls (p<0.001) in the three regions. The age distribution of schistosomiasis prevalence is shown in Figure 2. For statistical comparison, children were arbitrarily divided into three age groups (<9, 9–14 and ≥15 years). The results showed that the overall schistosomiasis prevalence was significantly higher in children aged nine years or above (p<0.05).

### Intensity of infection

**Figure 3:**
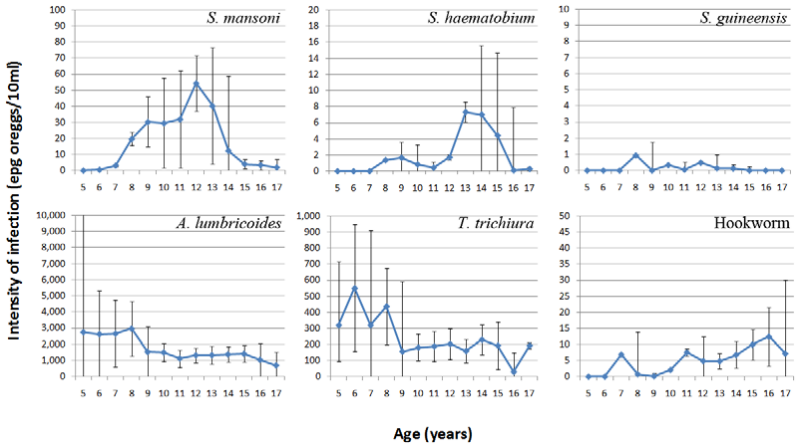
Arithmetic mean intensity of infection of schistosomiasis and STH by age in the surveyed regions. A few outliers below 5 or above 17 years old are not plotted. Error bars represent 95% CI.

The arithmetic mean intensity of infection in the three regions for each species of schistosomiasis is shown in Table 1. The egg counts for intestinal schistosomiasis ranged from 0 to 13,818 epg, and from 0 to 2,600 eggs/10 ml for urinary schistosomiasis. The overall arithmetic mean infection intensity was 33.24 epg for *S. mansoni*, 2.46 eggs/10 ml for *S. haematobium*, and 0.23 epg for *S. guineensis*. The Centre region was most heavily infected with *S. mansoni* (61.04 epg) and the West region with *S.haematobium* (6.86 eggs/10 ml). It appears that infections were light (<100 epg) in the majority of schools, with only 2.5% moderate or heavy *S. mansoni* infections and 0.72% heavy *S. haematobium* infections across the three regions (Table 1). Boys were more heavily infected with *S. mansoni* or *S. haematobium* than girls (p<0.01). The age distribution of intensity of infection for individual schistosome species is shown in Figure 3. Intensity of infection increased with age for *S. haematobium* in children examined while children of 9–14 years old were more heavily infected with *S. mansoni* (p<0.001).

### Soil-transmitted helminthiasis

#### Prevalence

**Figure 4:**
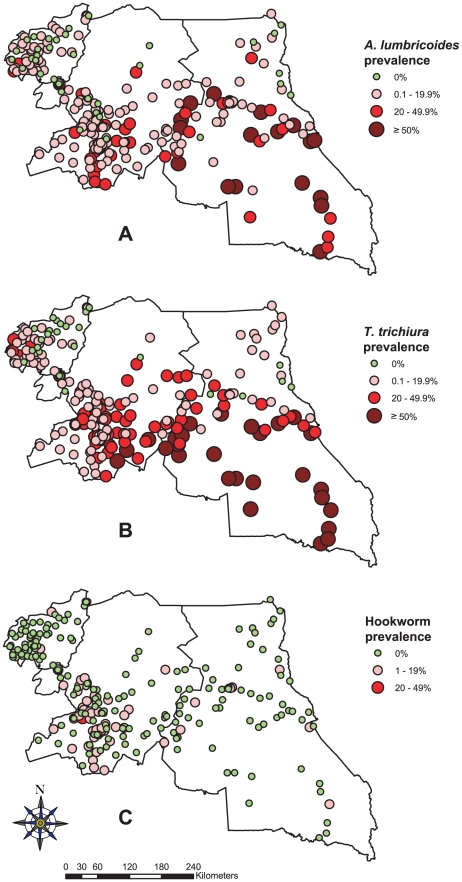
Prevalence of soil-transmitted helminthiasis by school in the three surveyed regions of Cameroon. (A) *A. lumbricoides*, (B) *T. trichiura*, and (C) Hookworm infections.

As shown in Table 1, *T. trichiura* was the most prevalent STH with an overall prevalence of 18.22%, followed by *A. lumbricoides* with an overall prevalence of 11.48% across the three regions, while hookworm prevalence was relatively low with only 1.55%. There was a significant geographical heterogeneity in STH distribution among schools surveyed across the three regions (Figure 4). The East region had the highest prevalence for both *T. trichiura* (37.52%) and *A. lumbricoides* (28.81%). There was not much difference in STH prevalence between boys and girls, though significantly different for *A. lumbricoides* and *T. trichiura*. As shown in Figure 2, *A. lumbricoides* and *T. trichiura* infections were more frequent in younger children aged below nine years (p<0.01), while hookworm infections increased with age (p<0.01).

The overall prevalence of STH (including all three species) across the three regions was 24.10%: 25.12% in the Centre region, 46.57% in the East region and 10.49% in the West region. There was significant difference between regions (p<0.001). Significantly more boys (25.63%) were infected than girls (22.58%) (p = 0.003). Children under nine years old were significantly more infected with STHs than older children (p<0.001), with prevalence of 35.56%, 23.25% and 24.83% for <9 years, 9–14 years and ≥15 years, respectively.

#### Intensity of infection

*A. lumbricoides* infection was the most heavy infection across the three regions, with an arithmetic mean intensity of 1418.23 epg, followed by *T. trichiura* infection with an arithmetic mean intensity of 199.15 epg, while hookworm infection was light (4.78 epg) (Table 1, Figure 3). The maximum egg count for *A. lumbricoides* was 518,688 epg, *T. trichiura* 122,400 epg, and hookworm 20,256 epg. In agreement with the prevalence data, the East region was most heavily infected with *A. lumbricoides* and *T. trichiura* (Table 1). Boys were slightly less heavily infected with *A. lumbricoides* but slightly more heavily infected with *T. trichiura* than girls (p<0.001) (Table 1). Younger children (below 9 years old) were more heavily infected with both parasites (p<0.001) while hookworm intensity of infection increased with age among children (p<0.05) (Figure 3).

### Comparison of 1985–1987 and 2010 data

**Figure 5:**
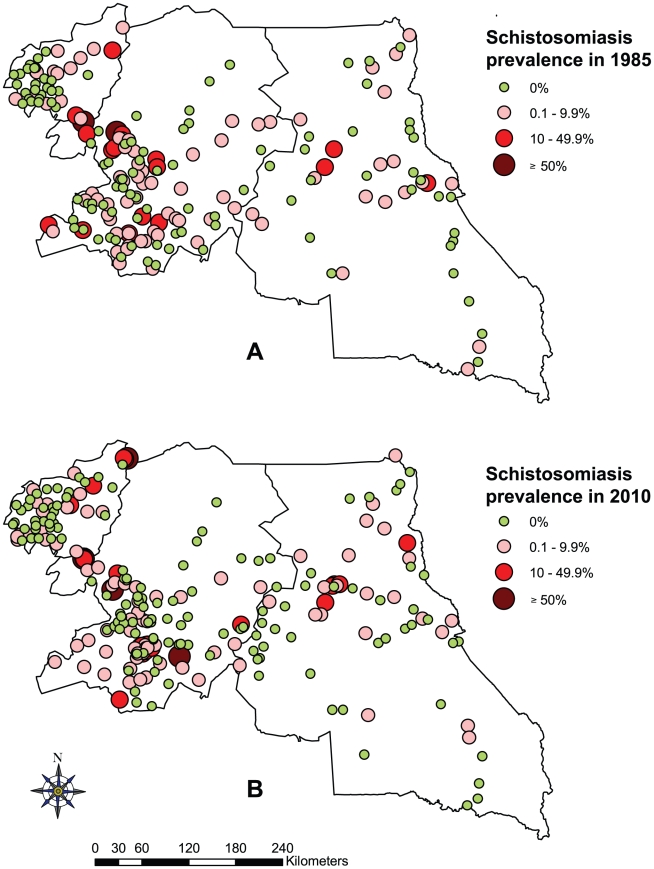
Comparative maps of the overall schistosomiasis prevalence in the three surveyed regions of Cameroon. Prevalence distribution in 1985–1987 (A) and in 2010 (B).

The current distribution of schistosomiasis and STH in 2010 was compared with the distribution in 1985–1987 [12]–[14], using the overall schistosomiasis and STH prevalence. The prevalence distribution of schistosomiasis in 1985–1987 and in 2010 is shown in Figures 5A and 5B, respectively, with the prevalence categorized according to the WHO prevalence thresholds [4]. It shows that the overall endemic areas of schistosomiasis did not change significantly. However, there was an increase in the number of high transmission foci of schistosomiasis in several health districts; e.g. health district of Malantouen in the West region where prevalence was up to 95.92% in the village of Matta, and health districts of Mbalmayo and Bafia in the Centre region with prevalence up to 71.43% and 52.78% in the villages of Dzeng and Yorro, respectively. Statistical comparison was carried out taking into account the geographical location of districts, age and sex. The results are shown in Table 2. Compared with the 1985–1987 data, the overall schistosomiasis prevalence in 2010 across the three regions and that in the Centre region did not change significantly (p>0.05), while prevalence in the East region decreased and prevalence in the West region increased, both significantly (p<0.01), though the level of infection in these two regions were relatively lower. Among the three schistosome species, the overall *S. haematobium* prevalence remained unchanged (p>0.05), while the overall *S. mansoni* prevalence significantly increased from 4.3% to 5.53% (p<0.05), and that of *S. guineensis*, though low, decreased significantly (p<0.001).

**Table 2:** Comparison of prevalence of schistosomiasis and STH between 1980s and 2010.

	Data from 1985–1987	Data in 2010	Percentage difference (%)
**Schistosomiasis prevalence (%)**			
Overall	6.28 (5.72–6.89)*	7.20 (6.76–7.65)	14.65
Centre	10.41 (9.26–11.68)	10.49 (9.80–11.23)	0.77
East	5.70 (4.71–6.88)	2.07 (1.55–2.79)	−63.68
West	2.44 (1.89–3.15)	3.97 (3.41–4.64)	62.70
**STH prevalence (%)**			
Overall	90.06 (89.45–90.63)	24.11 (23.37–24.86)	−73.23
Centre	93.02 (92.34–93.65)	25.12 (24.10–26.17)	−73.00
East	92.34 (91.00–93.49)	46.56 (44.39–48.72)	−49.58
West	81.14 (79.52–82.66)	10.51 (9.58–11.52)	−87.05
**Proportion of polyparasitic infections (%)**			
0 species	11.35 (10.61–12.14)	66.90 (66.05–67.74)	489.43
1 species	26.73 (25.67–27.81)	22.91 (22.16–23.67)	−14.29
2 species	49.89 (48.68–51.09)	9.58 (9.07–10.12)	−80.80
3 species	11.41 (10.67–12.20)	0.59 (0.46–0.74)	−94.83
4 species	0.61 (0.45–0.83)	0.02 (0.00–0.06)	−96.72
5 species	0.02 (0.00–0.09)	-	-

Note:

*Figures in brackets represent the 95% confidence intervals.

**Figure 6:**
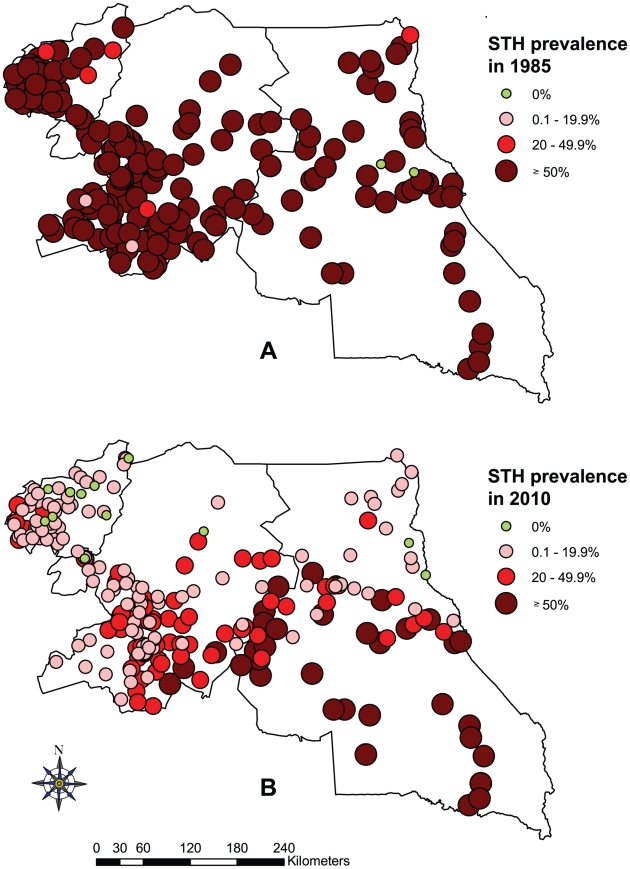
Comparative maps of the overall soil-transmitted helminthiasis prevalence in the three surveyed regions of Cameroon. Prevalence distribution in 1985–1987 (A) and in 2010 (B).

The prevalence distribution of STH in 1985–1987 and in 2010 is shown in Figure 6. There was a clear and significant decrease of STH prevalence in all three regions. Indeed, statistical comparison showed that the overall STH prevalence declined significantly from 93.02%, 92.34% and 81.14% to 25.12%, 46.56% and 10.51% in the Centre, East and West regions, respectively (all p<0.001) (Table 2). However, the decrease of STH was significantly lower in the East region in comparison to the two other regions. Detailed analysis of individual STH species showed significant reductions of 86.99% for hookworms, 82.27% for *A. lumbricoides* and 78% for *T. trichiura* (all p<0.001).

Analysis of polyparasitic infections showed that in 1985–1987, 61.93% of school-aged children examined were infected with more than one and up to five parasite species, but this proportion decreased significantly to 10.19% in 2010 (p<0.001) (Table 2).

## Discussion

The present study showed that schistosomiasis was moderately endemic (prevalence between 10–49%) in 23 of the 244 schools investigated, and highly endemic (prevalence ≥50%) in 4 schools. These moderate and high-risk communities are distributed in 13 of the 63 health districts investigated. The results confirmed the typical focal distribution of schistosomiasis in these regions. When comparing our results with the previous nationwide data collected in 1985–1987 by Ratard et al. [12], it appears a slight increase of the number of high transmission foci of schistosomiasis and an overall increase of *S. mansoni* infections – the most prevalent schistosome species in the three regions. This is not surprising given the fact that no MDA with praziquantel had been implemented in these health districts since the last mapping survey, apart from the health district of Ndikinimeki in the Centre region. The national control programme for schistosomiasis and intestinal helminthiasis was officially launched in 2004 in Cameroon [16]. Since 2007, school-aged children had been dewormed annually with mebendazole nationwide in all 179 health districts, whereas praziquantel were distributed only in schistosomiasis highly endemic health districts, including all 51 health districts of the three northern regions of Cameroon, where the highest transmission level of schistosomiasis were found [12], [29], and only in one of the 63 health districts of the three investigated regions, i.e. the district of Ndikinimeki, Centre region. The comparison of 1985 and 2010 data showed a significant decrease of schistosomiasis prevalence within the health district of Ndikinimeki, with a decline from 81.60% to 41% in the town of Makenene for example. Changing situation of schistosomiasis varied in the three regions and among the three different species, and this may reflect the differences in transmission dynamics in these different regions. The main factors influencing schistosomiasis transmission may include the changing demographic situation, socioeconomic development, water and sanitation, snail population dynamics etc. However, such information was not collected in the current mapping survey, which may be a topic for future studies.

One of the key outcomes and recommendations from this study is that in future deworming campaigns, the distribution of praziquantel should be undertaken in all 13 health districts in these three regions where schistosomiasis prevalence were ≥10%, according to WHO preventive chemotherapy guidelines [4]. Considering the overall low endemicity of schistosomiasis in the majority of these health districts, treatment will be conducted at district level in rural zones, whereas in urban settings treatment will be focused in those sub-districts with high prevalence spots of schistosomiasis. It should be noted that in both 1985 and 2010 surveys, single Kato-Katz slides were conducted as commonly used for mapping studies. Therefore, the prevalence and abundance of *S. mansoni* and STH may have been underestimated, due to the low sensitivity of Kato-Katz technique and day-to-day variation in egg excretions, particularly in light infections.

For STH, our study showed an overall significant decrease of infection prevalence in all three regions investigated, in comparison to previous mapping data collected in 1985–1987 [13], [14], [29]. Indeed, the STH prevalence declined from 93% to 25.1% in the Centre region, from 81% to 10.5% in the West region, and from 92.3% to 46.6% in the East region. These results clearly illustrate the positive impact of the school-based deworming campaigns with mebendazole implemented annually by the Ministry of Public Health, through the National Programme for the Control of Schistosomiasis and Intestinal Helminthiasis. The decline was lower in the East region compared to the two other regions. The previous mapping data showed that the three regions surveyed were among the higher STH prevalence areas within the country [13], [14]. Apart from the ivermectin MDA implemented in onchocerchiasis endemic communities, these regions have not been subjected to albendazole distribution which is used for lymphatic filariasis control. It is therefore interesting to see that the overall STH prevalence has been reduced so much by mainly mebendazole distribution. Though it has been shown that mebendazole is not as efficient as albendazole in deworming, particularly for hookworms [30]–[32], the present data show that mebendazole still has a significant role to play in the current effort to control NTDs. Several other factors, such as socio-economic development, improved sanitation and hygiene, environmental changes and collateral effect of other drugs, may have also contributed to the reduction of STH transmission. However, as discussed above this may be a topic for future studies.

Despite the observed significant reduction of STH infections, the prevalence and intensities of *A. lumbricoides* and *T. trichiura* infections were still relatively high, particularly in the East region. Several factors may explain the lower reduction of STH infections in this region, including the low socio-economic status and poor sanitation in most of the rural settings, which favor high parasite transmission and frequent human re-infections. The East region is the largest and the most sparsely populated region in Cameroon. The vast majority of its inhabitants being subsistence farmers, the low level of development in the region, and its thick forests and equatorial climate are favorable factors for STH and other NTDs. Also, the lower school attendance rates in villages, in comparison to towns, may have affected the treatment coverage of all school-aged children through a single school-based deworming campaign approach. It is well known that the epidemiology of STH infections is influenced by several determinants, including environment, population heterogeneity, age, household clustering, genetics and polyparasitism [33]. STHs affect the poor and infections are particularly abundant among people living in rural or deprived urban settings with low socio-economic status and poor sanitation [34]. Further investigations should be conducted to identify the major factors affecting the deworming effect in order to improve the impact of the current integrated NTD control programme.

The mapping results showed that the majority of health districts (34 over the total of 63, i.e. 53.97%) were still within the STH infection categories requiring large-scale preventive chemotherapy interventions, i.e. infection prevalence ≥20%. In communities with prevalence ≥50%, WHO recommends treatment of all school-aged children – enrolled and not enrolled – twice per year, and even three times if resources are available; whereas in communities where prevalence is ≥20% but <50%, school-aged children should be treated once a year. Therefore, the government of Cameroon should continue implementing annual deworming of school-aged children in all districts of the Centre, East and West regions. In addition, preschool children, women of childbearing age and adults at high-risk in certain occupations should also be treated, according to WHO recommendations [4]. In particular, in the East region where STH infection prevalence and intensities remain very high, it should be envisaged to deworm school-aged children at least twice a year. Furthermore, the alternating use of mebendazole and albendazole from one deworming round to another should be envisaged to optimize treatment efficacy against STHs [35].

Finally, the results of the present study highlight the new health districts where the MDA of praziquantel should be implemented for the treatment of schistosomiasis. For future deworming campaigns, all school-aged children should be treated with praziquantel in moderate (i.e. prevalence ≥10% but <50%) and high-risk communities (i.e. prevalence ≥50%). Also, praziquantel should be made available in dispensaries and clinics for treatment of suspected cases, in accordance with WHO recommendations [4]. Interestingly, this study provided data for accurate estimation of increased praziquantel needs, and the results will contribute to update global information on the distribution of schistosomiasis and STH, recently developed as an open-access database [36], [37].

## References

[1] Molyneux DH, Hotez PJ, Fenwick A (2005). “Rapid-impact interventions”: how a policy of integrated control for Africa's neglected tropical diseases could benefit the poor.. PLoS Med.

[2] Lammie PJ, Fenwick A, Utzinger J (2006). A blueprint for success: integration of neglected tropical disease control programmes.. Trends Parasitol.

[3] Utzinger J, de Savigny D (2006). Control of neglected tropical diseases: integrated chemotherapy and beyond.. PLoS Med.

[4] WHO (2006). Preventive Chemotherapy in human helminthiasis: coordinated use of anthelminthic drugs in control interventions.

[5] Hotez PJ, Molyneux DH, Fenwick A, Kumaresan J, Sachs SE (2007). Control of neglected tropical diseases.. N Engl J Med.

[6] Editorial (2007). A turning point for neglected tropical disease control.. Lancet.

[7] Grepin KA, Reich MR (2008). Conceptualizing integration: a framework for analysis applied to neglected tropical disease control partnerships.. PLoS Negl Trop Dis.

[8] Savioli L, Gabrielli AF, Montresor A, Chitsulo L, Engels D (2009). Schistosomiasis control in Africa: 8 years after World Health Assembly Resolution 54.19.. Parasitology.

[9] MINSANTE (2005). Programme National de Lutte contre la Schistosomiase et les Helminthiases Intestinales: Plan stratégique 2005–2010.

[10] Kane RA, Southgate VR, Rollinson D, Littlewood DT, Lockyer AE (2003). A phylogeny based on three mitochondrial genes supports the division of Schistosoma intercalatum into two separate species.. Parasitology.

[11] Pagès JR, Jourdane J, Southgate VR, Tchuem Tchuenté LA, Combes C, Jourdane J (2003). Reconnaissance de deux espèces jumelles au sein du taxon Schistosoma intercalatum Fisher, 1934, agent de la schistosomose humaine rectale en Afrique. Description de Schistosoma guineensis n. sp.. Taxonomy, Ecology and Evolution of Metazoan Parasites.

[12] Ratard RC, Kouemeni LE, Bessala MM, Ndamkou CN, Greer GJ (1990). Human schistosomiasis in Cameroon. I. Distribution of schistosomiasis.. Am J Trop Med Hyg.

[13] Ratard RC, Kouemeni LE, Ekani Bessala MM, Ndamkou CN, Sama MT (1991). Ascariasis and trichuriasis in Cameroon.. Trans R Soc Trop Med Hyg.

[14] Ratard RC, Kouemeni LE, Ekani Bessala MK, Ndamkou CN (1992). Distribution of hookworm infection in Cameroon.. Ann Trop Med Parasitol.

[15] Tchuem Tchuente LA, Behnke JM, Gilbert FS, Southgate VR, Vercruysse J (2003). Polyparasitism with Schistosoma haematobium and soil-transmitted helminth infections among school children in Loum, Cameroon.. Trop Med Int Health.

[16] Tchuente LA, N'Goran EK (2009). Schistosomiasis and soil-transmitted helminthiasis control in Cameroon and Cote d'Ivoire: implementing control on a limited budget.. Parasitology.

[17] Linehan M, Hanson C, Weaver A, Baker M, Kabore A (2011). Integrated implementation of programs targeting neglected tropical diseases through preventive chemotherapy: proving the feasibility at national scale.. Am J Trop Med Hyg.

[18] WHO (2010). Working to overcome the global impact of neglected tropical diseases.

[19] BUCREP (2010). 3e recensement général de la population et de l'habitat: La population du Cameroun en 2010.

[20] Ratard RC, Kouemeni L, Ekani Bessala MM, Ndamkou NC (1990). Distribution and preservation of Schistosoma mansoni eggs in stools.. J Trop Med Hyg.

[21] Montresor A, Crompton DWT, Hall A, Bundy DA, Savioli L (1998). Guidelines for the evaluation of soil-transmitted helminthiasis and schistosomiasis at community level.

[22] WHO (2010). Operational guide to mapping of schistosomiasis and soil transmitted helminthiasis and evaluation of control programmes.

[23] Hodges M, Dada N, Wamsley A, Paye J, Nyorkor E (2011). Improved mapping strategy to better inform policy on the control of schistosomiasis and soil-transmitted helminthiasis in Sierra Leone.. Parasit Vectors.

[24] Newcombe RG (1998). Two-sided confidence intervals for the single proportion: comparison of seven methods.. Stat Med.

[25] Fulford AJ (1994). Dispersion and bias: can we trust geometric means?. Parasitol Today.

[26] Montresor A (2007). Arithmetic or geometric means of eggs per gram are not appropriate indicators to estimate the impact of control measures in helminth infections.. Trans R Soc Trop Med Hyg.

[27] Toure S, Zhang Y, Bosque-Oliva E, Ky C, Ouedraogo A (2008). Two-year impact of single praziquantel treatment on infection in the national control programme on schistosomiasis in Burkina Faso.. Bull World Health Organ.

[28] WHO (2002). Prevention and control of schistosomiasis and soil-transmitted helminthiasis.. WHO Technical Report Series.

[29] Brooker S, Donnelly CA, Guyatt HL (2000). Estimating the number of helminthic infections in the Republic of Cameroon from data on infection prevalence in schoolchildren.. Bull World Health Organ.

[30] Albonico M, Smith PG, Hall A, Chwaya HM, Alawi KS (1994). A randomized controlled trial comparing mebendazole and albendazole against Ascaris, Trichuris and hookworm infections.. Trans R Soc Trop Med Hyg.

[31] Albonico M, Allen H, Chitsulo L, Engels D, Gabrielli AF (2008). Controlling soil-transmitted helminthiasis in pre-school-age children through preventive chemotherapy.. PLoS Negl Trop Dis.

[32] Keiser J, Utzinger J (2008). Efficacy of current drugs against soil-transmitted helminth infections: systematic review and meta-analysis.. JAMA.

[33] Hotez PJ, Brindley PJ, Bethony JM, King CH, Pearce EJ (2008). Helminth infections: the great neglected tropical diseases.. J Clin Invest.

[34] Hotez PJ, Bundy DAP, Beegle K, Brooker S, de Silva N, Jamison D, Claeson M, Breman J, Meacham A (2006). Helminth infections: soil-transmitted helminth infections and schistosomiasis.. Disease Control Pririoties in Developing Countries. 2nd ed.

[35] Tchuem Tchuenté LA (2010). Control of soil-transmitted helminths in sub-Saharan Africa: Diagnosis, drug efficacy concerns and challenges.. Acta Trop.

[36] Brooker S, Hotez PJ, Bundy DA (2010). The global atlas of helminth infection: mapping the way forward in neglected tropical disease control.. PLoS Negl Trop Dis.

[37] Schur N, Hurlimann E, Garba A, Traore MS, Ndir O (2011). Geostatistical model-based estimates of Schistosomiasis prevalence among individuals aged </ = 20 years in West Africa.. PLoS Negl Trop Dis.

